# Global Perspective on Fertility-Sparing Surgical Oncology in Gynaecology: A Narrative Literature Review

**DOI:** 10.7759/cureus.112012

**Published:** 2026-07-03

**Authors:** Mario Antonio Villatoro Bonilla, Roberth Josue Vera Tamayo, Paula Andrea González Tangarife, Isabel Híjar Cabello, Laura Ivette Casas Nava, Andrés Sebastián Estrella López

**Affiliations:** 1 Surgical Oncology, Clínica Dr. Mario Villatoro, San Salvador, SLV; 2 Faculty of Medicine, Pontificia Universidad Católica del Ecuador, Portoviejo, ECU; 3 Outpatient Care, Universidad de Manizales, Manizales, COL; 4 General Surgery, Hospital de Especialidades, Unidad Médica de Alta Especialidad (UMAE) No. 71, Torreón, MEX; 5 Obstetrics and Gynecology, Instituto de Seguridad y Servicios Sociales de los Trabajadores del Estado, Mexico City, MEX; 6 Public Health, NeoScientia Consulting Group, Quito, ECU

**Keywords:** cervical cancer, endometrial cancer, fertility preservation, fertility-sparing surgery, gynecologic oncology, neoadjuvant chemotherapy, ovarian cancer

## Abstract

Fertility-sparing surgery (FSS) has become a significant therapeutic option for young women with early-stage gynaecologic malignancies, aiming to balance effective cancer treatment with preservation of reproductive potential. However, the results are different based on the type of tumour, method of treatment, and mechanism of patient selection. This narrative literature review aimed to synthesise the current evidence on FSS oncology in gynaecology worldwide. The literature search included predominantly retrospective cohort studies, along with prospective cohort, cross-sectional, case-control, and randomised controlled studies, published between 1st January 2015 and 31st March 2026. The outcomes assessed were fertility preservation rates, pregnancy outcomes, live births, obstetric complications, oncologic outcomes (overall survival and recurrence), and prognostic factors affecting treatment success. Fertility preservation rates ranged from 55% to 100%, with rates exceeding 75% among patients with earlier-stage disease. Pregnancy outcomes were optimised in selected patients through the use of assisted reproductive technology (ART), including in vitro fertilisation (IVF). Conception occurred either spontaneously or through ART, with conception rates varying among women who attempted pregnancy. The live birth rates were between 20% and over 80%, but preterm delivery and obstetric complications were common. Across most included studies, the five-year overall survival rate exceeded 90% among patients with early-stage disease. Nevertheless, recurrence rates ranged from 1% to >20%, depending on tumour characteristics. Tumour size exceeding 2 cm, lymphovascular space invasion, and high-stage disease were consistently identified as significant predictors of recurrence and low disease-free survival. Current evidence suggests that FSS may be a safe and effective treatment option for carefully selected patients with early-stage gynaecological cancers while preserving reproductive potential. Fertility and survival outcomes should be optimised with the use of standardised protocols and long-term follow-up.

## Introduction and background

The global landscape of gynaecologic oncology is evolving as delayed childbearing, increasing obesity, and improvements in early cancer detection have contributed to a growing number of reproductive-aged women being diagnosed with gynaecologic malignancies [1]. Consequently, preserving fertility has become an increasingly important component of cancer care, as many patients wish to maintain their reproductive potential without compromising oncologic outcomes.

Cervical cancer remains the fourth most common cancer among women worldwide, with approximately one-third of patients diagnosed before the age of 40 years [2,3]. In China, approximately one in five women with cervical cancer is younger than 45 years at diagnosis, and nearly one-quarter have no children or only one child [4]. Similarly, approximately 3-17% of epithelial ovarian cancer cases occur in women of reproductive age [5,6], while approximately 3.2% of patients with endometrial cancer are younger than 40 years, the majority of whom are nulliparous [7]. These demographic trends highlight the growing need for fertility-preserving treatment strategies in appropriately selected patients.

Fertility preservation in gynaecologic oncology encompasses both surgical and selected nonsurgical treatment approaches, depending on the tumour type, stage, and patient characteristics. Fertility-sparing surgical procedures include cervical conisation, radical trachelectomy, unilateral salpingo-oophorectomy, ovarian cystectomy, and selected fertility-preserving staging procedures. In carefully selected patients with endometrial neoplasia, fertility preservation may also be achieved using hormonal therapy, with or without hysteroscopic resection, as hormonal management represents a nonsurgical alternative rather than a surgical intervention [8]. The primary objective of these approaches is to achieve effective oncologic treatment while preserving reproductive potential whenever oncologically appropriate.

Current international guidelines, including those from the European Society of Gynaecological Oncology (ESGO), the National Comprehensive Cancer Network (NCCN), and the European Society for Medical Oncology (ESMO), recommend fertility-sparing management only for carefully selected patients with early-stage disease following comprehensive counselling, accurate staging, and multidisciplinary evaluation. Although these guidelines support fertility preservation in selected cervical, ovarian, and endometrial cancers, they also acknowledge that many recommendations are based predominantly on retrospective observational studies and emphasise the need for individualised decision-making and long-term follow-up.

Growing evidence suggests favourable oncologic and reproductive outcomes following fertility-sparing management in carefully selected patients. For example, Gil-Ibanez et al. reported a three-year progression-free survival of 95.7% in patients with cervical tumours <2 cm undergoing radical trachelectomy, compared with 76.9% in those with tumours measuring 2-4 cm, with tumour size ≥2 cm identified as an important adverse prognostic factor [9]. Similarly, Ditto et al. demonstrated excellent oncologic outcomes following ultraconservative management of early-stage cervical cancer (IA2-IB1, <2 cm), with no recurrences reported and a five-year disease-free survival of 85.9% [10]. For patients with tumours >2 cm, neoadjuvant chemotherapy (NACT) before fertility-sparing surgery (FSS) has emerged as a potential strategy to expand eligibility. Rendón et al. reported a fertility preservation rate of 92% and a recurrence rate of 13% after a median follow-up of 47 months in carefully selected patients treated with NACT followed by FSS [11].

In epithelial ovarian cancer, FSS appears to be an acceptable option for selected patients with stage I disease, whereas malignant ovarian germ cell tumours, because of their high chemosensitivity, often permit fertility preservation even in selected advanced-stage cases without compromising survival [5,12]. Likewise, in carefully selected patients with grade 1, stage IA endometrioid endometrial carcinoma or atypical endometrial hyperplasia, hormonal therapy using oral progestins, levonorgestrel-releasing intrauterine systems, and gonadotropin-releasing hormone agonists has demonstrated encouraging reproductive and oncologic outcomes [1]. However, metabolic syndrome, obesity, polycystic ovary syndrome, and impaired glucose metabolism have been associated with reduced response rates and prolonged time to remission, highlighting the importance of individualised patient assessment before fertility-preserving treatment [7].

Despite encouraging outcomes, the available evidence remains predominantly retrospective and heterogeneous, with considerable variation in patient selection, surgical techniques, outcome reporting, and follow-up duration. These limitations make direct comparisons across studies difficult and underscore the need for a comprehensive synthesis of the current literature. Therefore, this narrative review aims to provide a global overview of fertility-sparing management in cervical, ovarian, and endometrial cancers by critically summarising the available evidence on oncologic safety, reproductive outcomes, and factors influencing treatment success, while also identifying current knowledge gaps and future research priorities.

One gap in the current literature on FSS in gynaecologic oncology is the lack of high-quality, prospective, and standardised evidence. Current international guidelines, including those from the ESGO, ESMO, and NCCN, support fertility-sparing management in carefully selected patients with early-stage gynaecologic malignancies. However, these recommendations are largely based on retrospective and heterogeneous evidence, highlighting the need for a comprehensive synthesis of the available literature. Furthermore, there is a lack of stratification of results by high-risk factors, such as tumours larger than 2 cm, nodal involvement, and aggressive histological types. Moreover, in cervical and endometrial cancers, the role of sentinel lymph node biopsy with ultra-staging, molecular profiling, and the optimal use of adjuvant therapy in selecting candidates for FSS remains an evolving area of research. There is poor reporting of reproductive outcomes, and they tend to be secondary to oncologic endpoints, providing an incomplete picture of fertility potential following treatment. Moreover, global differences in FSS accessibility and in approaches to surgery also reduce generalisability. These gaps together demonstrate the necessity of multicentre prospective research and international registries that are standardised and create evidence-based protocols to achieve a balance between oncologic safety and fertility preservation.

## Review

Methodology

This narrative literature review was conducted using a structured literature search to identify and qualitatively synthesise the available evidence on FSS in gynaecologic oncology. The review focused on oncologic, reproductive, and survival outcomes in patients with cervical, ovarian, and endometrial cancers. A structured search of PubMed, the Cochrane Library, and Google Scholar was performed to identify relevant studies published between 1 January 2015 and 31 March 2026. The search strategy incorporated Medical Subject Headings (MeSH) and free-text keywords related to FSS, fertility preservation, gynaecologic malignancies, cervical cancer, ovarian cancer, endometrial cancer, neoadjuvant chemotherapy, and hormonal therapy. The simplified search strategy is presented in Table 1.

**Table 1:** Search string

Database	Search String
PubMed	"fertility-sparing surgery" OR "fertility preservation surgery") AND ("cervical cancer" OR "ovarian cancer" OR "endometrial cancer") AND ("pregnancy outcome" OR "live birth") AND (recurrence OR survival)
Google scholar	"fertility-sparing surgery" AND "early cervical cancer" AND "live birth" AND recurrence AND survival
Cochrane	(fertility preservation OR fertility-sparing OR conservative management) AND (gynaecologic cancer OR cervical cancer OR ovarian cancer OR endometrial cancer OR uterine cancer) AND (pregnancy OR reproductive outcome OR fertility OR live birth OR obstetric outcome)

To enhance the transparency and comprehensiveness of the review, predefined eligibility criteria and a structured study selection process were applied. Titles and abstracts were screened for relevance, and potentially eligible articles underwent full-text review. Studies were included if they involved reproductive-age women undergoing fertility-sparing management for gynaecologic cancers and reported at least one of the following outcomes: oncologic outcomes (recurrence and survival), reproductive outcomes (pregnancy rate, live birth rate, miscarriage, and preterm delivery), or prognostic factors associated with treatment success.

Only full-text original research articles published in English were included. Cross-sectional, case-control, retrospective cohort, prospective cohort, and randomised controlled studies were eligible for inclusion. Reviews, systematic reviews, meta-analyses, editorials, conference abstracts, case reports, letters to the editor, and animal studies were excluded. Where appropriate, the reference lists of eligible studies were screened to identify additional relevant publications. Google Scholar was used as a supplementary search source to identify citation-linked articles and studies not captured through PubMed and the Cochrane Library. The exclusion of other major bibliographic databases, including Embase, Scopus, and Web of Science, was due to access and resource limitations and is acknowledged as a limitation of this review.

Although predefined eligibility criteria and a structured search strategy were employed to improve the transparency and reproducibility of evidence identification, this review was not conducted as a systematic review. Accordingly, formal risk-of-bias assessment, methodological quality appraisal, certainty-of-evidence evaluation, and quantitative meta-analysis were not performed, as the primary objective was to provide a qualitative synthesis of the current evidence rather than a formal evaluation of study quality. The findings were synthesised narratively and organised according to cancer type, fertility-sparing treatment approach, and reported oncologic and reproductive outcomes. As this review was based exclusively on previously published literature, ethical approval and informed consent were not required.

Selection Process of Studies

**Figure 1:**
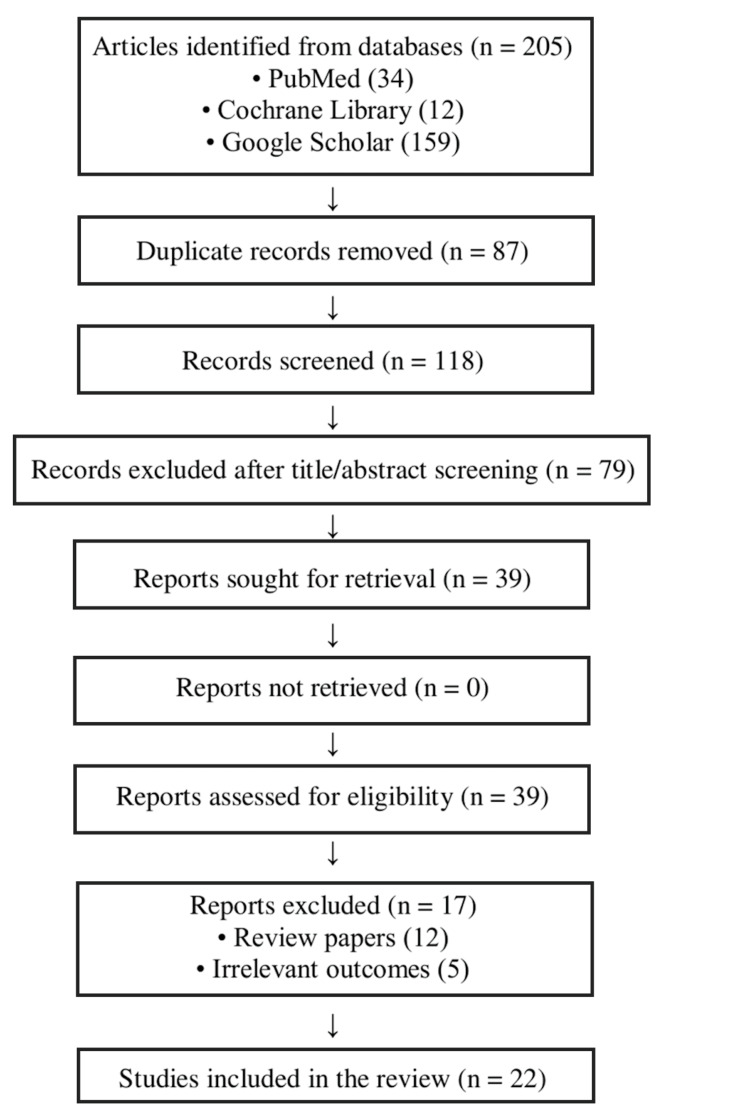
Study selection flow diagram

A total of 205 records were identified through database searching, including PubMed (n = 34), Cochrane Library (n = 12), and Google Scholar (n = 159). After the removal of 87 duplicate records, a total of 118 records remained for screening. Titles and abstracts were screened, and 79 records were excluded due to irrelevance to FSS or lack of relevant oncologic or reproductive outcomes, leaving 39 reports for full-text retrieval. All full-text articles were successfully retrieved and assessed for eligibility. During eligibility assessment, 17 studies were excluded, including 12 review articles and five studies that did not report relevant fertility or oncologic outcomes. Finally, 22 studies were included in the qualitative synthesis of this narrative literature review (Figure 1).

Synthesis of findings

The studies included are a diverse collection of observational cohorts, clinical trials, and case series assessing fertility-sparing strategies in gynaecologic malignancies, mostly low-stage cervical cancer, and ovarian and endometrial pathologies. The sample size was quite diverse: small case series (n=8) and large population-based studies (n=10,629) (Table 2). The majority of the cervical cancer literature is about young women of reproductive age (usually ≤40-45 years) who have a strong motivation to preserve their fertility and have the disease at an early stage (FIGO IA-IB), but some studies also dealt with tumours greater than 2 cm treated with neoadjuvant chemotherapy and FSS. Surgeries mostly involved fertility-sparing procedures, including conisation and simple or radical trachelectomy (through vaginal, abdominal, laparoscopic, or robotic surgeries), commonly accompanied by pelvic lymph node evaluation. Radical hysterectomy, non-radical procedures, or stratification by tumour size, histology, or neoadjuvant chemotherapy was used as a comparator, and some studies lacked direct comparison groups. The evaluation of the lymph nodes played a vital role in the majority of the cervical cancer research, and the node-negative condition was typically a precondition of fertility-sparing therapy; the incidences of nodal positivity were different among studies, ranging between 0 and about 27%. The most common subtypes in cervical cohorts included squamous cell carcinoma and adenocarcinoma with occasional adenosquamous and rare forms. The grade of the tumour was inconsistently reported, with most of the studies having a distribution of 1 to 3 and some having no significant impact on prognosis. Lymphovascular space invasion (LVSI) was sporadically reported, but when it occurred, it demonstrated inconsistent prevalence and a lack of prognostic value; some studies showed it to be a possible risk factor, while other studies did not find it to be a significant predictor of recurrence. In ovarian tumours, especially the malignant ovarian germ cell tumours and epithelial ovarian tumours, FSS usually includes the preservation of the uterus and the contralateral ovary, either with or without extensive staging. The assessment of lymph nodes was not always done and reported, and LVSI was not applicable in general. Likewise, in endometrial pathologies like atypical endometrial hyperplasia and early endometrial cancer, fertility-preserving treatment was based on hormonal therapies and no longer on surgical staging, so the evaluation of lymph nodes and LVSI was no longer applicable. Overall, the available evidence suggests that fertility-sparing management may be considered for carefully selected patients with well-staged, early-stage gynaecologic cancers. However, these findings should be interpreted with caution, as the available evidence is derived predominantly from retrospective studies with considerable heterogeneity in patient selection, surgical techniques, and outcome reporting. Furthermore, the inconsistent reporting of key pathological variables, including lymph node status, tumour grade, and LVSI, underscores the need for standardised outcome reporting and high-quality prospective studies (Table 3).

**Table 2:** Characteristics of the studies included in the literature review RCT = Randomised controlled trial; SEER = Surveillance, Epidemiology, and End Results database study; NCDB = National Cancer Database study; IPTW = Inverse Probability of Treatment Weighting; PSM = Propensity Score Matching; NA = Not applicable; NR = Not reported; ART = Assisted Reproductive Technology–preserving surgery; FSS = Fertility-Sparing Surgery; RH = Radical Hysterectomy; CRT = Chemoradiotherapy; LEEP = Loop Electrosurgical Excision Procedure; NACT = Neoadjuvant Chemotherapy; LEP = Laparoscopic Excision Procedure; LNG-IUS = Levonorgestrel-Releasing Intrauterine System; SCC = Squamous Cell Carcinoma; AC = Adenocarcinoma; ASC = Adenosquamous Carcinoma; AEH = Atypical Endometrial Hyperplasia; EC = Endometrial Cancer; EOC = Epithelial Ovarian Cancer; MOGCT = Malignant Ovarian Germ Cell Tumour; LVSI = Lymphovascular Space Invasion; LN = Lymph Node; G1 = Grade 1 (well differentiated); G2 = Grade 2 (moderately differentiated); G3 = Grade 3 (poorly differentiated); G3–4 = High-grade tumours. ART = abdominal radical trachelectomy; LEP = laparoscopic extraperitoneal pelvic lymphadenectomy. Surgical types are based on the Querleu–Morrow classification: Type C2 = nerve-non-preserving radical resection of the parametrium; Type D = laterally extended radical resection reserved for selected locally advanced disease.

Authors, Year	Study Design	Sample Size	Cancer Type	Population (P)	Intervention (I)	Comparison (C)	Lymph Node Status	Histology	Tumour Grade	LVSI Status
Bahrehmand et al. 2025 [2]	Retrospective cohort	18	Early-stage cervical cancer (>2 cm)	Women 24–40 years (mean 31.8), fertility desire	ART (Type C2 n=17; Type D/LEP n=1) + pelvic lymphadenectomy	Standard treatment (RH/CRT; refused)	Positive 27%; Negative 72%	Adenocarcinoma 50%; SCC 50%	G1 22%; G2 56%; G3 22%	Positive in all recurrences; none without LVSI recurred
Gil-Ibañez et al., 2022 [9]	Multicentre retrospective cohort	111	Early cervical cancer (IA1–IB1)	Women desiring fertility	Fertility-sparing surgery (FSS)	Tumour < 2 cm vs. 2–4 cm	Node-negative intraoperatively; 1 micrometastasis	SCC 59.5%; Adenocarcinoma 40.5%	Not reported	<2 cm: 8.2%; 2–4 cm: 40%
Slama et al., 2023 [3]	Multicentre retrospective cohort	733	Cervical cancer (IA1–II)	Women 18–40 years	FSS (conisation/trachelectomy)	Radical vs. non-radical surgery	Node-negative required	SCC 70.4%, AC 24.3%, and others 5.3%	G1–G3 (not significant)	Present in 49.9%; not significant
Tesfai et al., 2020 [13]	Retrospective cohort	19	Cervical cancer (>2 cm)	Women 19–36 years	NACT + ART + lymphadenectomy	Standard treatment if non-response	Mostly node-negative; 1 positive intra-op	SCC 74%, AC 21%, Clear cell 5%	Not reported	Not systematically reported
Ditto et al., 2015 [10]	Prospective cohort	22	Early cervical cancer (IA2–IB1)	Women ≤40 years	Conisation + pelvic lymphadenectomy	None	Node-positive 14%	SCC 45%; AC 50%; ASC 5%	G1 18%; G2 45%; G3 27%	41% positive
Wang et al., 2019 [4]	Multicentre prospective	83	Cervical cancer (IA1–IB1)	Women ≤40 years	Radical/subradical trachelectomy + lymphadenectomy ± chemo	Surgical and chemo comparisons	No metastasis (5 excluded)	SCC 93.9%; AC 6.0%	G1 44.6%; G2 37.3%; G3 18.1%	8.4% positive
Ditto et al., 2024 [14]	Retrospective cohort (IPTW)	109	Early cervical cancer	Women 18–44 years	FSS + lymph node evaluation	Radical hysterectomy	FSS 10.2% positive; RH 5% positive	SCC ~55%; AC ~41%; ASC ~2–3%	G1–G3 similar between groups	FSS 38.8%; RH 36.7%
Valzacchi et al., 2023 [15]	Retrospective cohort	48	Early cervical cancer (IA2–IB1)	Women desiring fertility	Conisation or radical trachelectomy	Surgical approach comparison	Node-negative; no parametrial disease	SCC 56.2%; AC 39.6%; ASC 4.2%	G1 47.9%; G2 45.8%; G3 6.3%	18.75% positive
Ning et al., 2024 [16]	Retrospective (SEER)	10,629	Cervical cancer (Stage I)	Women 15–39 years	FSS	Radical hysterectomy	Not reported	SCC 64.9%; AC 29.9%; others	G1–2 47.4%; G3–4 19.6%	Not reported
Llueca et al., 2022 [17]	Multicentre retrospective (PSM)	222	Early cervical cancer	Women with fertility desire	FSS	Radical hysterectomy	Node-negative; 1 micrometastasis	SCC ~62%; AC ~38%	Not reported	~14–18% positive
Rendón et al., 2021 [11]	Multicentre retrospective	25	Cervical cancer (≥2 cm)	Women <40 years	NACT + FSS	None	Mostly node-negative; 1 positive	SCC 92%; AC 8%	G1 24%; G2 56%; G3 20%	Not reported
Plaikner et al., 2023 [18]	Multicentre retrospective	31	Cervical cancer (>2 cm)	Women ~29 years	NACT + vaginal trachelectomy	None	All node-negative	SCC 65%; AC 32%; ASC 3%	G1 10%; G2 54%; G3 36%	23% positive
Yang et al., 2016 [19]	Retrospective cohort	106	MOGCT	Women's median is 22 years	Fertility-preserving surgery	Radical surgery	LN metastasis ~18%	Mixed germ cell types	Not reported	Not applicable
Nasioudis et al., 2017 [12]	Retrospective (NCDB)	526	Advanced MOGCT	Women <40 years	Fertility-preserving surgery	Hysterectomy	LN dissection ~75–83%	Dysgerminoma 42%; others	Not reported	Not applicable
Lee et al., 2020 [20]	Retrospective cohort	32	Cervical cancer (IA1–IB1)	Women <45 years	Conisation/LEEP or trachelectomy	Procedure comparison	5% node-positive (IB1)	SCC predominant	Not reported	IA1: 0%; IB1: 80%
Kajiyama, Yoshihara, et al., 2019 [21]	Multicentre retrospective	78	Ovarian EC (Stage I)	Women <45 years	FSS	Radical surgery	Not reported	Endometrioid	Mostly G1–2	Not applicable
Gama et al., 2023 [22]	Case series	8	Synchronous tumours	Women 28–34 years	Hormonal + ovarian surgery	None	Node-negative	Low-grade tumours	Low grade	Not reported
Kajiyama, Suzuki, et al., 2019 [5]	Multicentre retrospective	285	Epithelial ovarian cancer	Women ≤45 years	FSS	Radical surgery	Not specified	WHO types	Not detailed	Not reported
Watanabe et al., 2020 [6]	Multicentre retrospective	29	Stage I EOC	Women ≤40 years	FSS	None	Not reported	Mucinous-predominant	G1–G3	Not reported
Li et al., 2024 [23]	Retrospective cohort	173	AEH/early EC	Women ≤45 years	Progestin therapy	Time-to-response groups	Not applicable	AEH/EC	Not detailed	Not applicable
Ding et al., 2022 [7]	Retrospective cohort	106	AEH/early EC	Women <45 years	Hormonal therapy	Metabolic comparisons	Not applicable	Endometrioid G1	G1 only	Not applicable
Goh et al., 2024 [1]	RCT (Phase II)	36	AEH	Women 21–40 years	Megestrol acetate	LNG-IUS	Not applicable	AEH	Not applicable	Not applicable

**Table 3:** Findings of the studies included in the literature review RCT = Randomised controlled trial; SEER = Surveillance, Epidemiology, and End Results database study; NCDB = National Cancer Database study; IPTW = Inverse Probability of Treatment Weighting; PSM = Propensity Score Matching; NA = Not applicable; NR = Not reported; ART = Assisted Reproductive Technology–preserving surgery; FSS = Fertility-Sparing Surgery; RH = Radical Hysterectomy; RS = Radical Surgery; CRT = Chemoradiotherapy; NACT = Neoadjuvant Chemotherapy; IVF = In Vitro Fertilisation; CR = Complete Response; SCC = Squamous Cell Carcinoma; AC = Adenocarcinoma; ASC = Adenosquamous Carcinoma; ER/PR = Oestrogen Receptor/Progesterone Receptor; AEH = Atypical Endometrial Hyperplasia; EC = Endometrial Cancer; EOC = Epithelial Ovarian Cancer; MOGCT = Malignant Ovarian Germ Cell Tumour; LVSI = Lymphovascular Space Invasion; LN = Lymph Node; FIGO = International Federation of Gynecology and Obstetrics staging system; OS = Overall Survival; RFS = Recurrence-Free Survival; G1 = Grade 1 (well differentiated); G2 = Grade 2 (moderately differentiated); G3 = Grade 3 (poorly differentiated).

Authors, Year	Fertility Preservation Rate	Patients Attempting Conception	Mode of Conception	Live Births	Preterm Delivery Rate	Nodal Metastasis (Successful Case)	Follow-Up (Months)	Overall Survival	Recurrence Rate	Key Prognostic Factor
Bahrehmand et al., 2025 [2]	77.8%	64.3%	IVF only	2	50%	Yes (1 case)	Median 143.3	5-year OS 93.3%	16.7% overall; 7% FSS	LVSI; tumour >2 cm without LVSI favourable
Gil-Ibañez et al., 2022 [9]	100%	26.1%	Not reported	Not clearly reported	~35%	No	30.7–55.7	Not fully reported	9.9% overall	Tumour size ≥2 cm
Slama et al., 2023 [3]	Not reported	Not reported	Not reported	Not reported	Not reported	Not reported	Median 72	Mortality 2.6%	7.0%	Tumour size >2 cm
Tesfai et al., 2020 [13]	74%	20%	Spontaneous	6	0%	No	Median 50	5-year RFS 82%	15.7%	Tumour response to NACT; histology
Ditto et al., 2015 [10]	100%	83%	Spontaneous	5	7%	No	Mean: 48.8	5-year OS 93.7%	9.1%	Nodal status; tumour size
Wang et al., 2019 [4]	94.3%	83.1%	Spontaneous	50	13.8%	No	Median 36.2	Not reported	1.2%	Tumour size >2 cm
Ditto et al., 2024 [14]	79.6%	69.4%	Spontaneous	12	Low (~1 case)	No	Median 38.8	No difference vs RH	12.2% (FSS)	Site of relapse; cervical recurrence salvage
Valzacchi et al., 2023 [15]	100%	60.4%	Spontaneous	~88% of pregnancies	~33%	No	Median 66	5-year OS 96%	12.5%	Tumour size
Ning et al., 2024 [16]	24.5%	Not reported	Not reported	Not reported	Not reported	Not reported	Not reported	5-year OS ~94–97%	Not reported	Stage, grade, histology
Llueca et al., 2022 [17]	100%	Not reported	Not reported	Not reported	Not reported	No	~63–66	No noninferiority vs RH	9.9–28.9%	Tumour size >2 cm
Rendón et al., 2021 [11]	92%	43.5%	Not specified	11 births	63.6%	No	Median: 47	100% survival	13%	Tumour response; nodal status
Plaikner et al., 2023 [18]	87%	67%	Spontaneous	12	100%	No	Median 94.5	96%	11.1%	Residual tumour; tumour >2 cm
Yang et al., 2016 [19]	55.7%	Not clearly defined	Not specified	33	Not reported	Not applicable	Median 56.5	5-year OS 66%	10.4%	Residual tumour; chemotherapy cycles
Nasioudis et al., 2017 [12]	79.8%	Not reported	Not reported	Not reported	Not reported	Not applicable	Median 57	5-year OS ~94%	Not reported	Stage; histology
Lee et al., 2020 [20]	100%	36.7%	Not specified	11	54.5%	Yes (1 case)	~49 months	Not clearly reported	12.5%	Tumour size; LVSI; margins
Kajiyama, Yoshihara, et al., 2019 [21]	30.7%	Not reported	Not reported	Not reported	Not reported	Not reported	Median 65.3	5-year OS ~96%	11.5%	FIGO stage
Gama et al., 2023 [22]	87.5% CR	Not reported	IVF	4	Not clearly defined	Not applicable	Median 50.5	100%	0%	Patient selection; ER/PR status
Kajiyama, Suzuki, et al., 2019 [5]	35.4%	Not reported	Not reported	Not reported	Not reported	Not reported	Median 66	No difference vs. RS	14.7%	FSS not prognostic
Watanabe et al., 2020 [6]	100%	Not reported	Not specified	6	Not reported	Not applicable	Median 60.6	5-year OS ~96%	17.2%	FIGO stage; cytology
Li et al., 2024 [23]	Not applicable	Not reported	Not reported	Not reported	Not reported	Not applicable	Not reported	Not reported	Not reported	Metabolic risk score
Ding et al., 2022 [7]	100%	Not reported	Not reported	Not reported	Not reported	Not applicable	~35–42	Not reported	~18–37%	Metabolic syndrome
Goh et al., 2024 [1]	88.9% CR	59.4%	Mixed (spontaneous + IVF)	4	High miscarriage rate	Not applicable	Median 22.5	100%	17.2%	Obesity; metabolic factors

Fertility Preservation Rate

Across the included studies, fertility preservation rates were generally high, particularly among carefully selected patients with early-stage gynaecologic malignancies, supporting the feasibility of fertility-sparing management in appropriate clinical settings. Preservation in cervical cancer was as high as around 74-100%, with Bahrehmand et al. (77.8%) and Ditto et al. (79.6%) showing uterine conservation in most of the patients [2,10]. Preservation rates were found to be near-universal in carefully selected cohorts undergoing FSS. This indicates that, with strict patient selection, almost all eligible patients were able to preserve reproductive function after treatment [9,10,15]. Lower preservation rates (30-55%) in ovarian malignancies were observed due to stricter patient selection and more cautious oncologic considerations. This reflects the need to balance fertility preservation with ensuring long-term oncologic safety in higher-risk disease settings [19,21]. These results underscore that fertility preservation is highly feasible in early-stage disease when performed with the right patient selection, underscoring its position as a standard practice in contemporary gynaecologic oncology.

Conception and Mode of Pregnancy

The mode of pregnancy and the method of conception vary across studies and are influenced by patient intent, length of follow-up, and counselling. The proportion of patients attempting conception ranged from 20% to 83%, reflecting desire in reproductive desires and clinical guidance. Both spontaneous and assisted reproductive methods were employed all over the world. In most studies, including Wang et al., where 58 pregnancies were all spontaneous [4], and Ditto et al., where 58-91% of conceptions were all natural [14]. On the other hand, assisted reproductive technologies (ART), especially in vitro fertilisation (IVF), played a vital role in the populations in which all pregnancies were obtained by IVF [2,22]. This difference highlights the need to consider reproductive medicine as part of the oncologic care pathways to maximise fertility.

Live Birth Outcomes

Fertility preservation is not limited to anatomical preservation but also to functional reproductive success, as live births were favourable among patients who conceived. Several studies have reported high live birth outcomes, with Wang et al. (50 live births) and Yang et al. (33 live births) having the highest live birth outputs [4,19]. Smaller groups were also significant in their results, like Ditto et al. (five live births) and Gama et al. (four live births) [10,22]. Significantly, the outcomes of neonatal care were mostly positive, and most infants were reported as healthy at birth [4,6]. These results help to prove the clinical significance of FSS, and it is possible to state that successful delivery and carrying a pregnancy are achievable.

Obstetric Outcomes and Preterm Delivery

Although fertility rates have increased overall, preterm birth remains a major concern in current research on fertility preservation outcomes. Reported rates vary widely depending on patient selection and treatment history. In low-risk, carefully selected cohorts, preterm birth rates as low as 0% have been reported [13], whereas in high-risk groups, particularly those undergoing neoadjuvant chemotherapy and radical trachelectomy, rates have reached as high as 100% [18]. Moderate preterm birth rates have also been observed, including 13.8% in Wang et al. and 54.5% in Lee et al. [4,20]. These results are indicative of structural compromise of the cervix and treatment-related variables, indicating the importance of special obstetric monitoring. Worldwide, this highlights the fact that fertility preservation is not only possible but also obstetric risks need to be well addressed.

Fertility Preservation in Nodal Metastasis

Fertility preservation in the presence of lymph node metastasis remains uncommon and highly controversial. Most studies have excluded node-positive patients from FSS protocols because of significant oncologic safety concerns [17]. The available evidence is limited to isolated case reports and should be regarded as anecdotal rather than generalisable. For example, Bahrehmand et al. reported a node-positive patient who achieved a live birth following IVF and remained disease-free at a five-year follow-up [2]. Similarly, Lee et al. described a node-positive patient who underwent adjuvant chemotherapy and achieved long-term disease-free survival [20]. Although these reports suggest that favourable outcomes may be possible in exceptionally selected patients, they are insufficient to support routine fertility-sparing management in node-positive disease, and further prospective evidence is required.

Survival Rate

Oncologic safety outcomes of fertility-sparing strategies have generally been favourable, with overall survival (OS) rates remaining high, particularly in early-stage disease. Across the included observational studies, reported OS rates generally exceeded 90%, including 93.3% in Bahrehmand et al., 93.7% in Ditto et al., and 96.0% in Valzacchi et al. [2,14,15]. In addition, larger comparative analyses have demonstrated no significant difference in survival between fertility-sparing and radical surgical approaches [12,16]. Similar findings have also been observed in ovarian malignancies, where fertility preservation did not appear to compromise survival outcomes [16,18]. Overall, these results strongly support the oncologic safety of fertility-sparing approaches in appropriately selected patients, reinforcing their potential broader implementation in clinical practice.

Recurrence Rates

Recurrence rates varied considerably depending on tumour characteristics, treatment approach, and patient selection. The lowest recurrence rates were observed in well-selected patient groups, with figures as low as 1.2% [4]. In contrast, higher-risk subsets, particularly those with larger tumours or more advanced disease, showed substantially increased recurrence, reaching up to 28.9% [17]. Regularly, tumour size more than 2 cm proved to be one of the key factors influencing the risk of recurrence in various research studies [3,9,17].

Key Prognostic Factors

A number of prognostic factors have been consistently identified in the literature as influential in patient selection and clinical outcomes worldwide. The most robust predictor of both recurrence and poorer survival is a tumour size greater than 2 cm [3,9,17]. Some studies have indicated that LVSI was an independent predictor of recurrence, particularly in larger tumours [2,20]. Other issues included histological subtype (non-squamous subtypes were associated with worse outcomes), tumour grade, and neoadjuvant chemotherapy response [13,19]. Residual tumour burden and FIGO stage were the predominant prognostic factors in ovarian cancer [6,19], and metabolic factors affected outcomes in endometrial disease [7,23]. These results support that fertility-sparing management in gynaecologic oncology is not only possible but also oncologically safe in well-selected patients, especially those with small disease. Although the reproductive outcomes are promising, differences in obstetric risks and recurrence highlight the importance of multidisciplinary care, strict selection criteria, and extended follow-up. This promotes the incorporation of fertility preservation into routine oncologic care and encourages further development of selection criteria and outcome reporting.

Critical Comparison of Findings

The comparative analysis of the studies included indicates consistency and heterogeneity of the outcomes concerning the FSS methods. In most studies, the fertility preservation rate exceeded 75%, with early-stage cancers and strict selection criteria [2,4,14]. In contrast, population-based analyses like Ning et al. (2024) reported lower rates due to broader inclusion criteria and real-world variability in clinical decision-making [16]. Reproductive success was significantly different according to tumour type, treatment modality, and assisted reproduction. Some studies indicated that they were highly reliant on IVF [2,22], whereas other studies showed successful spontaneous conception [4,15]. The live birth rates were favourable, but the rates of preterm birth were quite high, especially in women who had undergone radical trachelectomy or received neoadjuvant chemotherapy [11,18].

The results of oncologic processes were generally encouraging, with five-year OS in early-stage disease often exceeding 90%. Nonetheless, the recurrence rates were not consistent, with the lowest rates of 1.2% and the highest rate of more than 20% in patients who had tumours over 2 cm [4,9]. Tumour size was consistently the strongest prognostic variable, with tumours 2 cm or larger associated with worse outcomes [12]. Other risk factors were LVSI, lymph node metastasis, and poor adherence to treatment [20].

Limitations

The literature review is limited by the predominance of retrospective, single-centre studies with heterogeneous study designs, patient selection criteria, surgical methods, and outcome reporting, which limit the ability to directly compare studies. Moreover, the reproductive outcomes, live birth, and pregnancy complications were not consistently reported, and they were frequently considered secondary outcomes. Clinical heterogeneity is further enhanced by the fact that these gynaecologic malignancies are diverse in nature and have different disease stages, which limits the interpretation of findings. The literature search was restricted to PubMed, the Cochrane Library, and Google Scholar. Consequently, relevant studies indexed exclusively in other major bibliographic databases, such as Embase, Scopus, or Web of Science, may not have been identified. This may have reduced the comprehensiveness of the evidence base and resulted in the omission of potentially relevant studies, thereby limiting the completeness of the narrative synthesis. Although Google Scholar was used to enhance the comprehensiveness of the search by identifying additional and citation-linked studies, its limitations in terms of reproducibility and indexing transparency should be acknowledged. As this study was conducted as a narrative literature review, formal risk-of-bias assessment, methodological quality appraisal, certainty-of-evidence evaluation, and quantitative meta-analysis were not performed. This represents an important limitation of the review, as the methodological quality and risk of bias of the included studies could not be formally evaluated. Consequently, the strength and certainty of the synthesised evidence and the conclusions drawn from it should be interpreted with appropriate caution. Therefore, the findings represent a qualitative synthesis of the available evidence rather than an evidence-graded systematic assessment.

Future recommendations

Future research should prioritise well-designed, multicentre prospective cohort studies and international collaborative registries using standardised protocols for patient selection, surgical techniques, follow-up, and outcome reporting. Although randomised controlled trials may provide high-level evidence in selected clinical settings, they are often neither feasible nor ethically appropriate in fertility-sparing surgical oncology. International registries should be established to facilitate the standardised collection of oncologic and reproductive outcomes, including long-term survival, recurrence, fertility preservation, pregnancy rates, live births, and obstetric outcomes. Particular attention should be directed toward high-risk patient groups, including those with tumours >2 cm, lymph node-positive disease, and aggressive histological subtypes, to better define appropriate selection criteria for fertility-sparing management. Future studies should also investigate the role of molecular biomarkers, sentinel lymph node mapping, and ART in developing individualised fertility-sparing strategies that optimise both oncologic safety and reproductive outcomes.

## Conclusions

Current evidence suggests that FSS is a feasible option for carefully selected patients with early-stage gynaecologic cancers, offering favourable reproductive outcomes while appearing to maintain acceptable oncologic safety. However, these findings should be interpreted with caution, as the available evidence is predominantly retrospective, heterogeneous, and supported by limited long-term prospective data. Careful patient selection, accurate staging, multidisciplinary management, and close follow-up remain essential. Future research should prioritise multicentre prospective cohort studies and international registries with standardised outcome reporting to strengthen the evidence base and optimise individualised fertility-sparing management.
